# Laboratory Evaluation of a Quaternary Ammonium Compound-Based Antimicrobial Coating Used in Public Transport during the COVID-19 Pandemic

**DOI:** 10.1128/aem.01744-22

**Published:** 2023-03-01

**Authors:** Paz Aranega-Bou, Natalie Brown, Abigail Stigling, Wilhemina D’Costa, Neville Q. Verlander, Thomas Pottage, Allan Bennett, Ginny Moore

**Affiliations:** a Biosafety, Air and Water Microbiology Group, United Kingdom Health Security Agency, Salisbury, United Kingdom; b Statistics, Modelling and Economics Department, United Kingdom Health Security Agency, United Kingdom; Centers for Disease Control and Prevention

**Keywords:** SARS-CoV-2, antimicrobial coating, QAC, public transport, COVID-19, ɸ6

## Abstract

The virucidal activity of the Zoono Z71 Microbe Shield surface sanitizer and protectant, a quaternary ammonium compound (QAC)-based antimicrobial coating that was used by the United Kingdom rail industry during the COVID-19 pandemic, was evaluated, using the bacteriophage ɸ6 as a surrogate for SARS-CoV-2. Immediately after application and in the absence of interfering substances, the product effectively reduced (>3 log_10_) the viability of ɸ6 on some materials that are typically used in rail carriages (stainless steel, high-pressure laminate, plastic). If, after the application of the product, these surfaces remained undisturbed, the antimicrobial coating retained its efficacy for at least 28 days. However, efficacy depended on the material being coated. The product provided inconsistent results when applied to glass surfaces and was ineffective (i.e., achieved <3 log_10_ reduction) when applied to a train arm rest that was made of Terluran 22. Regardless of the material that was coated or the time since application, the presence of organic debris (fetal bovine serum) significantly reduced the viricidal activity of the coating. Wiping the surface with a wetted cloth after the deposition of organic debris was not sufficient to restore efficacy. We conclude that the product is likely to be of limited effectiveness in a busy, multiuser environment, such as public transport.

**IMPORTANCE** This study evaluated the performance of a commercially available antimicrobial coating that was used by the transport industry in the United Kingdom during the COVID-19 pandemic. While the product was effective against ɸ6, the efficacy of the coating depended upon the material to which it was applied. Similarly, and regardless of the surface material, the presence of organic debris severely impaired viricidal activity, and efficacy could not be recovered through wiping (cleaning) the surface. This highlights the importance of including relevant materials and conditions when evaluating antimicrobial coatings in the laboratory. Further efforts are required to identify suitable infection prevention and control practices for the transport industry.

## INTRODUCTION

Surfaces can become contaminated with virus via direct contact with body fluids or soiled hands and via the deposition of aerosolized virus. Contaminated surfaces can act as fomites and contribute to the spread of viral infections (1), and, while fomite transmission is not currently believed to be the primary transmission route for SARS-CoV-2 (2), the importance of this pathway in relation to other pathways might differ in different venues and situations (3). Although the risk of transmission following brief contact with a single contaminated surface is estimated to be low, the risk increases as individuals touch a higher number of contaminated surfaces (2). Factors, such as the number of contaminated hand contact sites, the density of individuals, and the duration of stay within a venue, along with the efficacy of any mitigation strategies, such as surface decontamination and handwashing, influence the risk of the transmission of viruses via fomites (2, 3). The use of public transport can require an individual to touch or grip a number of surfaces, including poles, seat head rests, tables, push buttons, and arm rests.

Despite usage remaining considerably lower than pre-pandemic levels, during the 2020 to 2021 financial year, 388 million rail passenger journeys were made in Great Britain, and this number increased to 990 million during 2021 to 2022 (4). In England alone, 1.57 billion local bus passenger journeys were made during 2021 to 2022 (5). Public transport use and accessibility are associated with better air quality, higher rates of employment, and lower social exclusion, and they can encourage a more active lifestyle (6). However, the role that public transport plays in the transmission of infectious diseases is not well-understood, although there is some epidemiological evidence that it could contribute to the transmission of influenza-like illness (7, 8), and high-touch surfaces on public transport vehicles are known to be contaminated with bacteria (9) and can be contaminated with SARS-CoV-2 RNA (10, 11). A recent modeling study that compared the relative contributions of close-range exposure to SARS-CoV-2 (via droplets or aerosols), airborne exposure (via small aerosols without having to be within 2 meters of the infectious source), and exposure via contaminated fomites in a subway carriage concluded that all three routes of transmission are relevant in this setting (12).

The field of antimicrobial coatings has developed rapidly in recent years, and their uses (in conjunction with handwashing and cleaning) as a potential infection prevention strategy by which to reduce health care-associated infections have been described (13). The COVID-19 pandemic has demonstrated the need to apply infection prevention and control principles in public spaces outside of health care, such as public transport. However, guidelines are scarce, and more research is needed to understand what practices are appropriate for the transport sector.

In the United Kingdom, some transport operators, in addition to having implemented enhanced cleaning protocols, have used antimicrobial coatings in an attempt to reduce the surface bioburden and the risk of viral contamination. There are a number of commercially available products that can be applied to existing surfaces and, according to their manufacturers’ claims, provide long-lasting residual antimicrobial activity when they are used in conjunction with regular cleaning (14). The US Environmental Protection Agency (US EPA) has developed an interim method that outlines the requirements for the registration of antimicrobial coatings that are intended to provide residual antimicrobial activity for a period of weeks and are applied to surfaces to supplement standard disinfection practices. The protocol includes an efficacy assessment after coated surfaces are subjected to cycles of dry and wet abrasion, using specialized equipment to simulate wear (15). However, this equipment is not widely available, and, in most cases, the manufacturer’s claims are supported by limited evidence that was often obtained through laboratory tests that did not attempt to mimic real-life conditions. Here, we used the bacteriophage ɸ6 as a surrogate for SARS-CoV-2 to evaluate the antiviral efficacy of the Zoono Z71 Microbe Shield surface sanitizer and protectant (Zoono, Bury St. Edmunds, United Kingdom), which has been used by the transport industry throughout the pandemic.

## RESULTS

### Efficacy testing on coupons.

**FIG 1:**
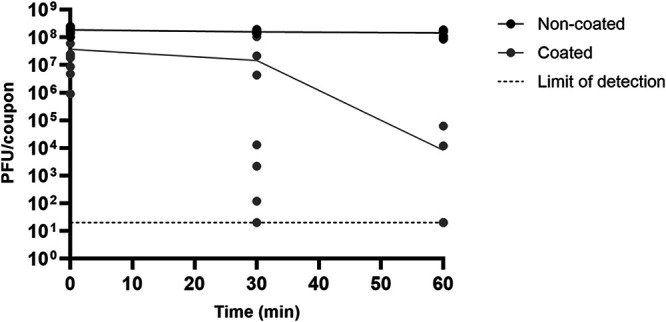
Survival of ɸ6 over 60 min on glass coupons coated with Zoono Z71 (gray circles) and noncoated coupons (black circles) that were processed in parallel. Coupons were coated by either spraying (*n* = 2 experimental trials) or pipetting 75 μL on each coupon (*n* = 1 experimental trial). All conditions were tested in triplicate on the three experimental trials, and individual results are expressed as PFU per coupon (PFU/coupon). The individual dots represent the results for individual coupons, and the lines show the mean for each time point. No ɸ6 could be recovered from 3 out of the 9 coated coupons after 30 min or from 7 out of the 9 coated coupons after 60 min. The concentrations of these samples were assumed to be 20 PFU/coupon, which is the theoretical limit of detection, for the analysis.

Under laboratory conditions and in the absence of interfering substances, Zoono Z71, when applied to glass, achieved reductions in ɸ6 viability that ranged from 0.7 to 4.5 log_10_ and from 3.9 to 6.9 log_10_ values over 30-minute and 60-minute contact times, respectively (*n* = 3 experimental trials). While the recovery of ɸ6 from coated glass coupons was variable (Fig. 1), the losses in ɸ6 viability on noncoated coupons were minimal (0.0 to 0.2 log_10_ values), indicating that ɸ6 was stable on the coupons over the course of the experiment and that natural losses in viability were not the reason for the variability observed (Fig. 1). The concentration of ɸ6 that was recovered from the coated glass coupons was statistically lower than that which was recovered from noncoated coupons. How the coating was applied (manual spraying versus pipetting) did not significantly impact the ɸ6 recovery (Table S3).

In contrast, Zoono Z71 consistently achieved ≥6.6 log_10_ reductions within a 60-minute contact time when applied to stainless steel or polystyrene coupons, irrespective of the number of days since application (Table 1). Over 120 min, the log_10_ reduction on noncoated coupons ranged from −0.1 to 0.3 log_10_ values. When the efficacy was assessed after a shorter contact time (5 min) the median (interquartile range [IQR]) reduction in the number of ɸ6 recovered from the coated polystyrene and stainless steel coupons equated to 3.6 (3.4 to 4) and 1.4 (0.8 to 1.8) log_10_ values, respectively. After 15 min, the reductions were ≥6.5 (≥6.3 to ≥6.5) and ≥6.5 (≥6.2 to ≥6.6) log_10_ values. Under the experimental conditions described (no disturbance of the coupons or interfering material present), neither the material type (stainless steel or polystyrene) nor the number of days since the application of the coating had a significant effect on efficacy (Table S4).

**TABLE 1:** Efficacy of Zoono Z71 when applied to stainless steel or polystyrene surfaces and inoculated with ɸ6^*a*^

Days after application of antimicrobial coating	Stainless steel		Polystyrene	
	Contact time			
	60 min	120 min	60 min	120 min
1	≥6.9	≥6.9	≥7.0	≥6.9
7	≥6.9	≥6.8	≥6.7	≥6.9
14	≥6.9	≥6.9	≥6.8	≥6.9
21	≥6.8	≥6.6	≥6.9	≥6.6
28	≥7.0	≥6.7	≥7.0	≥7.1

a Efficacy is expressed as the mean log_10_ reduction (*n* = 1 experimental trial with three coupons per condition), calculated by subtracting the mean ɸ6 PFU recovered from coated coupons from the mean ɸ6 PFU recovered from noncoated coupons at each contact time. Undetected virus observations were assumed to have a concentration of 20 PFU/coupon, which is the theoretical limit of detection, for the analysis.

### Evaluation of BSA and FBS as interfering substances.

To mimic the accumulation of organic material on surfaces that is present in busy spaces, such as public transport, a layer of BSA or FBS was applied to coated and noncoated coupons, prior to the inoculation of ɸ6. When FBS was applied over the antimicrobial coating, the recovery of ɸ6 was significantly higher than that observed when BSA or no interfering material was applied, with no significant difference between the latter two conditions (Table S5). Although a >3 log_10_ reduction within 60 min was achieved (Table 2), the presence of FBS had a greater impact at shorter contact times. The reduction of ɸ6 on coated coupons was monitored over time and, in the presence of FBS, ranged from 0.3 to 1.1 log_10_ values within 30 min. In contrast, ≥6 log _10_ reductions were observed when ɸ6 was applied to coated coupons without FBS under the same conditions. The presence of an FBS layer did not impact ɸ6 survival on the noncoated coupons, with reductions of 0.1 to 0.2 log_10_ values being observed over 120 min.

**TABLE 2:** Efficacy of Zoono ZT1 when applied to stainless steel and polystyrene test coupons coated with a layer of BSA or FBS prior to the inoculation of ɸ6^*a*^

Experimental conditions	Stainless steel		Polystyrene	
	Contact time			
	60 min	120 min	60 min	120 min
BSA experiment
Clean	≥6.8	≥6.6	≥7.1	≥7.0
BSA	≥6.9	≥6.8	≥7.0	≥7.1
FBS experiment
Clean	≥6.8	≥6.7	≥7.1	≥6.9
FBS	4.5	4.6	4.0	4.7

a Efficacy is expressed as the mean log_10_ reduction (*n* = 1 experimental trial with three coupons per condition), calculated by subtracting the mean ɸ6 PFU recovered from coated coupons with or without an interfering substance from the mean ɸ6 PFU recovered from noncoated coupons with or without the same interfering substance at each contact time. Undetected virus observations were assumed to have a concentration of 20 PFU/coupon, which is the theoretical limit of detection, for the analysis.

### Efficacy testing on train parts.

The efficacy of Zoono Z71 was also tested on a train tray table, arm rest, and hand pole. Based on previous results, the efficacy of the antimicrobial coating was evaluated in the presence or absence of FBS only. When the results for the tray table and arm rest were analyzed, there was a significant three-way interaction between the presence of the product, the presence of FBS, and the surface to which the product was applied. The tray table was comprised of two different materials (high-pressure laminate [HPL] [side A] and coated stainless steel [CSS] [side B]). In the absence of FBS, the average log_10_ reduction that was associated with the antimicrobial coating was significantly higher on side B, compared to side A. The addition of FBS significantly reduced the efficacy of Zoono Z71, regardless of the material (Table S6). The efficacy of the antimicrobial coating, when applied to HPL (side A), and in the absence of interfering material, ranged from 4.3 to >6.9 log_10_ values within a contact time of 120 min. The presence of FBS reduced the efficacy of the coating with log_10_ reductions not exceeding 1.1 over the same contact period. Similarly, when the coating was applied to CSS (side B) with and without FBS, the concentration of ɸ6 after 120 min was reduced by <1.6 and ≥6.1 log_10_ values, respectively, compared to the noncoated controls (Table 3). The overall recovery of ɸ6 after 120 min was lower from the Terluran 22 arm rest, compared to that observed with other materials, but the application of the antimicrobial coating did not lead to significant reductions of the ɸ6 concentration on this material (Table S6). Log reductions of <0.5 log_10_ values were observed, regardless of the contact time or the presence/absence of FBS, with the recovery of ɸ6 sometimes being higher in the presence of the antimicrobial coating, compared to the noncoated controls (Table 3).

**TABLE 3:** Efficacy of Zoono Z71 when applied to train parts^*a*^

Days after coating was applied	Experimental conditions	Tray table (side A, HPL)	Tray table (side B, CSS)	Arm rest (Terluran 22)	Hand pole (plastic coated)
		Contact time			
		120 min	120 min	120 min	120 min
≤7	Clean	≥6.9	≥6.8	−0.4	≥6.1
	FBS	1.1	1.6	0.1	0.8
≤7	Clean			-0.1	
	FBS			0.1	
>7 and ≤14	Clean	4.8	≥7.0	−1.0	
	FBS	0.1	0.5	0.5	
>14 and ≤21	Clean	5.6	≥6.7		≥6.3
	FBS	0.5	0.4		0.8
>21 and ≤28	Clean	4.3	≥6.1	−1.5	
	FBS	0.2	0.1	−0.4	

a Surfaces were coated with a layer of FBS prior to the inoculation of ɸ6. Each row represents an experimental trial, with three test areas (containing one droplet of ɸ6 each) being analyzed per condition. The efficacy is expressed as the mean log_10_ reduction, calculated by subtracting the mean ɸ6 PFU recovered from coated surfaces with or without FBS from the mean ɸ6 PFU recovered from noncoated surfaces with or without FBS after 120 min. Undetected virus observations were assumed to have a concentration of 20 PFU/replicate, which is the theoretical limit of detection, for the analysis. No data are available for cells shaded in gray. Side A of the tray table was tested 6, 12, 19, and 25 days after coating. Side B of the tray table was tested 4, 11, 17, and 26 days after coating. The arm rest was tested 4, 5, 13, and 27 days after coating. The hand pole was tested 7 and 20 days after coating.

The results for the hand pole had to be analyzed independently, as a different inoculum size was used, due to structural constraints. A significant interaction was found between the presence of the antimicrobial coating and the presence of FBS. As with the tray table, the presence of FBS did not significantly affect the recovery of ɸ6 from the hand pole when no antimicrobial coating was present (Tables S6 and 7). However, in comparison to the noncoated control, the application of the antimicrobial coating resulted in a significant reduction in ɸ6 recovery after a contact time of 120 min (≥6.1 log_10_ reductions) but not in the presence of FBS (0.8 log_10_ reduction) (Table 3; Table S7).

Even in the absence of FBS, the antimicrobial coating was less effective over shorter contact times. After 15 min (and in comparison to *t* = 0), the median (IQR) reduction of ɸ6 on the coated tray table and hand pole (*n* = 7 experimental trials) equated to 0.8 (0.2 to 0.9) log_10_ values.

**FIG 2:**
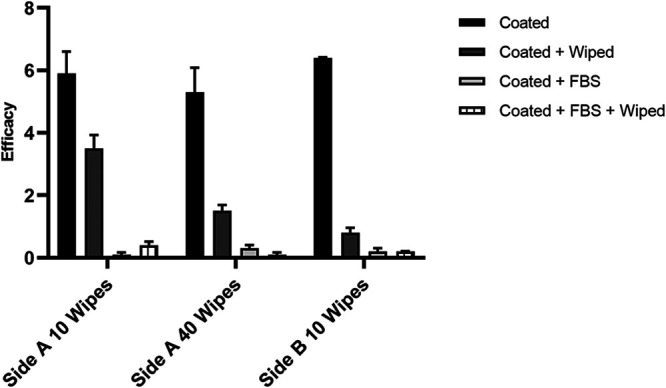
Efficacy of Zoono Z71 within a 120-minute contact time, when applied to a tray table (Side A, HPL; Side B, CSS), in the presence or absence of FBS, after wiping with a wetted cloth, in comparison with nonwiped controls. Two wiping protocols that differed in the number of wipes were applied. The efficacy is expressed as the mean log_10_ reduction, calculated by subtracting the mean ɸ6 PFU recovered on coated tray tables with or without FBS and with or without wiping from the mean ɸ6 PFU recovered from the matched noncoated control tray tables, after 120 min of contact time in two independent experimental trials with three test areas (containing one droplet of ɸ6 each) being analyzed per condition. The error bars represent the standard deviation. Undetected virus observations were assumed to have a concentration of 20 PFU/replicate, which is the theoretical detection limit, for the analysis.

The next study determined whether contaminating organic debris could be removed from a surface to restore the efficacy of the product. Coated and noncoated tray tables with and without FBS were wiped with a cloth that was wetted with sterile water, prior to the inoculation of ɸ6. A significant interaction was found between the application of the antimicrobial coating, the presence of FBS, and whether the surface had been wiped (Table S8). As seen previously, when FBS was present, the efficacy of the product was depleted (<0.3 log_10_ reduction over 120 min). Wiping the surfaces with a wetted cloth after the application of FBS did not restore the efficacy of the coating (Fig. 2). When no interfering substance was present, wiping significantly reduced the efficacy of the coating (Table S8). In the absence of FBS, the antimicrobial coating achieved log_10_ reductions of 5.9 (side A) and ≥6.4 (side B) within 120 min. Following the 10-wipe protocol, the log_10_ reductions achieved were 3.5 (side A) and 0.8 (side B). Increasing the number of wipes to 40 further reduced the efficacy of the coating applied to side A from 5.3 (in the absence of wiping) to 1.5 log_10_ values (Fig. 2).

## DISCUSSION

Environmental surfaces can become contaminated by a range of pathogenic microorganisms and can contribute to the spread of infectious diseases, including respiratory viruses, such as SARS-CoV-2. Strategies to prevent or reduce surface contamination, such as hand hygiene, the use of face coverings, and effective cleaning and disinfection, can prevent transmission through this route (16, 17). Antimicrobial coatings are an attractive strategy by which to supplement cleaning and maintain low levels of surface contamination between cleaning episodes, but evidence of efficacy with which to support their implementation is lacking.

Quaternary ammonium compound-based disinfectants are widely used in industrial, health care, and domestic settings (18), and they have been shown to be effective against SARS-CoV-2 (19). They are cationic detergents that present with a wide variety of chemical structures that are suitable for different applications, including polymer-based coatings, and they have been shown to inactivate bacteria, yeast, and viruses (18). In this study and in previous studies, when assessed under laboratory conditions and in the absence of interfering substances, quaternary ammonium polymer-based coatings, when applied to stainless steel, polystyrene, acrylonitrile butadiene styrene (ABS) plastic, and poly(methyl methacrylate) coupons (14, 20–23), have often shown high virucidal efficacy against SARS-CoV-2 and its surrogates (ɸ6 and human coronavirus 229E). However, it is worth noting that low efficacy has also been reported for some QAC-based products, even those with the same or similar active ingredients as those that were previously shown to be effective (14, 22), suggesting that different formulations of QAC-based antimicrobial coatings do not necessarily share the same efficacy profile.

In this study, we extended the testing to other materials that are frequently used in the transport industry and observed different levels of efficacy. Whereas the product was effective when applied to a plastic-coated hand pole and to a tray table comprised of both HPL and CSS surfaces, results were highly variable when it was applied to glass, and little to no virucidal activity was observed when the coating was applied to a Terluran 22 arm rest. The manufacturer claims that the coating is effective on all materials, but these results suggest that this is not the case. The testing of antimicrobial coatings should incorporate a range of surface materials, as efficacy might differ among them. Hardison et al., 2021 (22) also observed high variability between replicates for one of the products that they tested as well as differences in the efficacy of another product when it was applied to stainless steel and ABS plastic. Although it has been claimed that QAC that is immobilized on a surface inactivates bacteria and viruses on contact (24), this does not mean that the effect is immediate. In this study, and in the absence of interfering substances, contact times of at least 15 min were often required to achieve >3log_10_ reductions.

Public transport is a busy, multiuser environment in which organic debris frequently deposit on surfaces. This scenario was simulated by incorporating a layer of BSA or FBS over the coating. BSA is a protein that is commonly used in disinfectant testing (19, 25), and it is the major component of FBS, which has also been previously used as an organic challenge (22, 23, 26). FBS has many more components, including lipids and carbohydrates, and it is therefore likely to be more representative of general organic debris that are present on surfaces within public transport. In this study, to simulate the accumulation of organic debris on surfaces, the interfering substances were applied as a layer over surfaces that had previously been coated with the antimicrobial, as opposed to adding the organic load to the viral suspension. Whereas BSA did not interfere with the product, when a layer of FBS was applied to the test coupons, virucidal activity was reduced. When FBS was applied to the coated train parts, the virucidal activity of the product was eliminated. Different application methods (the coupons were coated via pipetting, whereas the train parts were coated via fogging) or differences in the materials might explain the discrepancy between the reduction of virucidal activity on the coupons and the elimination on the train parts. In agreement with these results, FBS has previously been reported to diminish the virucidal activity of a benzalkonium chloride-based liquid disinfectant against equine herpesvirus type 1 (26).

As the product is designed to be used alongside regular cleaning, we also investigated whether wiping to remove the FBS from the tray table could restore the efficacy of the coating on this surface. When FBS was present, wiping with a wetted cloth was not able to restore the efficacy of the product. When FBS was not present, wiping with a wetted cloth reduced the virucidal activity of the product, suggesting that the coating was being removed from the surface by the mechanical action of cleaning. Whether FBS had permanently inactivated the antimicrobial coating or whether the coating was more effectively removed from the surface in the presence of FBS is unknown. Previous studies have found similar issues with the durability of QAC-based antimicrobial coatings. Butot et al. (21) reported the loss of antiviral activity of a QAC-based antimicrobial coating after only one round of cleaning with a microfiber cloth and a water-based detergent or disinfection with 70% ethanol. Calfee et al. (14) found that seven QAC-based formulations that were effective against ɸ6 on initial testing were completely ineffective, following exposure to wet abrasion cycles in accordance with US EPA interim guidance (15). Other laboratory studies evaluating QAC-based products have not attempted to assess the durability (20, 22, 23).

This study has several limitations. First, although the tests were conducted using train parts that were taken from a scrap train and FBS was used to simulate organic debris, the experiments were conducted under laboratory conditions that do not fully represent a real-life setting. While every effort has been made to identify the materials of the train parts used in this study, they are not completely accurate, and they are unlikely to be representative of all materials used in public transport. Moreover, testing was done using the bacteriophage ɸ6 as a surrogate for SARS-CoV-2. ɸ6 is an enveloped dsRNA phage of the *Cystoviridae* family that shares structural features with SARS-CoV-2; it is enveloped by a lipid membrane, has spike proteins, is of a similar size (approximately 80 to 100 nm) (27, 28), and has been previously used as a surrogate for coronaviruses in studies evaluating environmental persistence and disinfection efficacy (28–31). However, the suitability of ɸ6 as a surrogate depends on the specific experimental conditions (29). Therefore, the results in this report might not accurately represent the behavior of SARS-CoV-2. Any materials that are associated with the virus (cell debris, soil, fluids) are also known to impact the disinfection efficacy (32). Here, we used TSB as a matrix (instead of respiratory fluids/sweat) for the viral suspension and also applied FBS to the surface (instead of the organic debris that are found on public transport), which might have also influenced the results. High viral titers were used in this study, and it is unknown whether the virucidal activity would differ at lower titers. Finally, the ɸ6 suspension was applied as a 5 to 10 μL droplet, which is representative of large droplet contamination but may not accurately represent the deposition of virus for all contamination scenarios (e.g., by touch, smaller droplets, or aerosols). Previous work with bacteria reported reduced efficacy for copper alloys when the inoculum was applied as an aerosol under reduced relative humidity (40%), as opposed to a wet inoculum (33). Therefore, it is possible that the efficacy of the product has been overestimated for some contamination scenarios in this study.

In conclusion, this study highlights the importance of testing QAC-based antimicrobial coatings on a range of materials in the presence of relevant interfering substances and assessing durability in order to decide whether their application will reduce the surface contamination burden in a given setting. For this particular product, the results suggest that efficacy in public transport surfaces will likely be quickly diminished due to the accumulation of organic debris, which is inevitable in a busy, multiuser environment. Given that wiping the dirty surfaces with a wetted cloth failed to restore efficacy, it is unlikely that the application of this product will provide a substantial benefit in this setting. More research is warranted to identify suitable infection prevention and control practices for public transport.

## MATERIALS AND METHODS

### Virus propagation.

Bacteriophage ɸ6 (DSM 21518) and host bacteria Pseudomonas syringae (Pseudomonas sp. [DSM 21482]) were obtained from the DSMZ GmbH, where they had been deposited by S. Moineau from Université Laval, Québec. P. syringae was reconstituted according to the manufacturer’s instructions, stored on cryobeads (Technical Service Consultants, Heywood, United Kingdom) at −80°C, and cultured weekly on tryptone soya agar (TSA) (Oxoid Ltd., Basingstoke, United Kingdom). ɸ6 was stored at 4°C (for no longer than 5 months) and propagated in P. syringae host cells.

A 10 μL loopful of P. syringae was transferred from TSA to 10 mL tryptone soya broth (TSB; E&O Laboratories Ltd., Bonnybridge, United Kingdom) and incubated for 18 to 20 h (25°C; 150 rpm). 100 μL of the resulting suspension were transferred to 10 mL TSB and incubated at 25°C (170 rpm). When the turbidity of the broth culture (OD_600_) exceeded 0.1 (approximately 5 h), 50 μL of ɸ6 suspension (either provided as a liquid suspension by DSMZ [unknown concentration] or the previously propagated working stock [approximately 2 × 10^10^ PFU/mL]) were added. Cultures were incubated overnight (25°C; 150 to 170 rpm) until total lysis. Propagated phage was filtered using a 0.22 μm PES syringe filter (Starlab, Milton Keynes, United Kingdom), and the concentration was determined via plaque assay. Phage suspensions (approximately 2 × 10^10^ PFU/mL) were stored at 4°C for up to 5 months.

### Preparation and coating of test coupons.

Glass coupons (1.56 cm^2^) were cut from microscope slides (Marienfeld, Lauda-Königshofen, Germany) using a glass cutter. Polystyrene coupons (2.54 cm^2^) were bought from Amazon (Fingooo or Suloli brands). Stainless steel coupons (1.44 cm^2^) were obtained from Apsley Precision Engineering Ltd. (Salisbury, United Kingdom). Glass and stainless steel coupons were washed with 5% Decon 90 (Decon, Sussex, United Kingdom) for 5 min, rinsed with demineralised water and 70% isopropanol (IPA), allowed to dry, and then autoclaved (121°C for 15 min). Polystyrene coupons were disinfected with 70% IPA and left to dry. The ready-to-use antimicrobial coating product Zoono Z71 Microbe Shield surface sanitizer and protectant was tested in this study. The product information sheet lists the quaternary ammonium compound alkyl (C12-16) dimethylbenzyl ammonium chloride (ADBAC/BKC [C12-16]) CAS 68424-85-1 (commonly known as benzalkonium chloride) at a concentration of 0.1% (wt/wt) as the active ingredient of Zoono Z71. Glass coupons were initially coated by manual spraying, but, due to low reproducibility, it was decided to pipette 75 μL of the product onto each coupon and to spread it evenly over the surface with the pipette tip. Stainless steel and polystyrene coupons were coated by pipetting 40 μL per coupon. The product was applied approximately 24 h before inoculation, unless otherwise stated.

### Preparation and coating of train parts.

**FIG 3:**
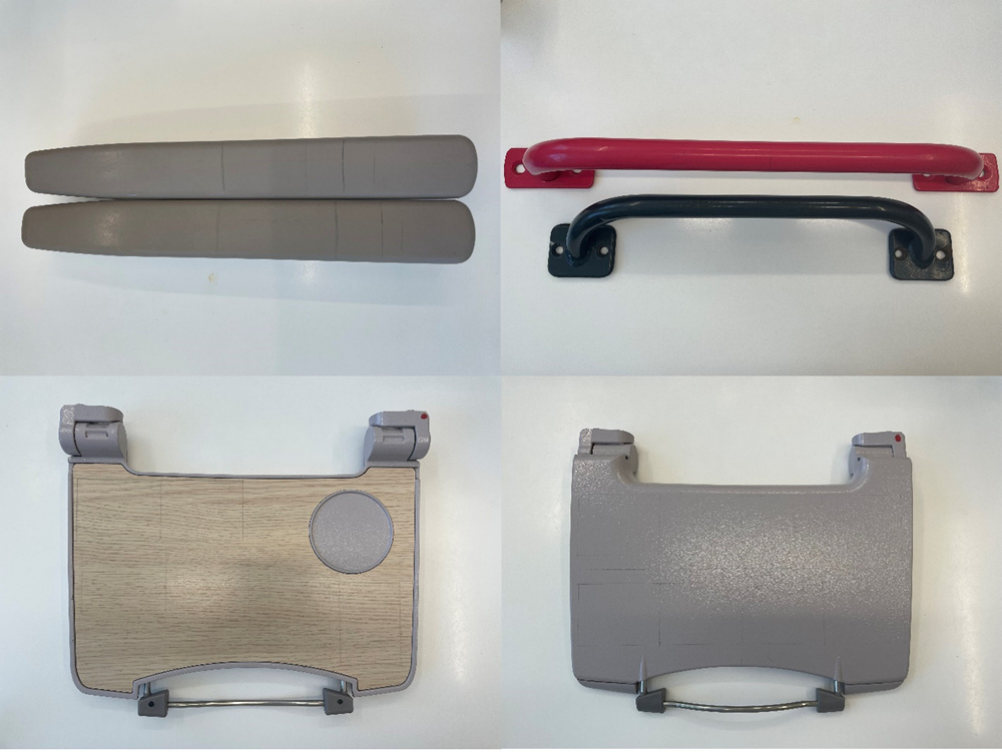
Top left, Terluran 22 arm rests; top right, plastic coated hand poles; bottom left, HPL tray table (Side A); bottom right, CSS tray table (Side B).

Previously used train parts were donated by First Group (Aberdeen, United Kingdom) from a scrap train. These consisted of tray tables made of high-pressure laminate (HPL) and coated stainless steel (CSS), arm rests made of Terluran 22, and hand poles with a plastic coating (Fig. 3). Upon receipt, the train parts were cleaned and disinfected by wiping with 70% IPA wipes (Sani-Cloth) until the wipes were not visibly dirty after the surface was wiped. Between experiments, the train parts were cleaned with 70% IPA and a Kimtech 7644 wiper, prior to a further neutralization step that used Dey-Engley neutralizing broth (DEB) (Sigma-Aldrich/Merck), and then rinsed with sterile water. The last two steps were to ensure that the antimicrobial coating that was previously applied had been neutralized and removed. The coating was applied using an electrostatic sprayer (Comac E-Spray) to ensure a homogenous distribution. The train parts were placed in a Class III cabinet, with the front panel removed and the fans on, while the spraying was undertaken. The train parts were sprayed from a distance of approximately 0.15 to 0.3 m until the surfaces were thoroughly wetted, and they were then allowed to dry overnight within the cabinet, without the fan operating. The surfaces were coated up to 28 days prior to inoculation to assess the claims that efficacy is retained for 30 days post-application.

### Efficacy testing.

Coated and noncoated test surfaces (material coupons and train parts) were inoculated with 10 μL droplets of a ɸ6 suspension (approximately 2 × 10^8^ PFU), with the exception of the hand pole, which was inoculated with 5 μL droplets due to structural constraints (approximately 1× 10^8^ PFU). All conditions were tested in triplicate for each experimental trial. Surfaces were sampled either immediately (*t* = 0) or after a predetermined contact time of up to 120 min under ambient laboratory conditions. At each time point, individual coupons (*n* = 3 for each material) were transferred to tubes containing 2 mL DEB and 4 glass beads. The train parts were sampled using FLOQSwabs (Copan Diagnostics, Murrieta, USA). Three test areas, containing one droplet each, were swabbed at each time point. Each swab was placed in 2 mL DEB that contained 4 glass beads. The suitability of DEB to neutralize the antimicrobial coating in liquid form was assessed before any efficacy tests were carried out (34). The tubes were vortexed for 30 s and were serially diluted in TSB before the concentration of ɸ6 was determined via plaque assay. The recovery efficiencies from the noncoated train parts and the control coupons at *t* = 0 were >90% and >95%, respectively.

### Plaque assay.

Soft phage agar (0.6%; Media Services, UKHSA) in 3 mL aliquots was melted (by heating to 100°C), maintained at 60°C, and cooled (37°C, 5 min) before being mixed with 250 μL *P syringae* (grown in TSB; OD_600_ > 0.9) and 100 μL of test suspension. The soft phage agar was then poured over the surface of a TSA plate and evenly distributed. Once the soft agar layer had set, the plates were incubated at 25°C for 18 to 24 h, and the resulting plaques were manually enumerated. The assays were conducted in duplicate and included control samples containing no ɸ6 to monitor for contamination and to ensure appropriate lawn formation by P. syringae. Validation work showed that the samples that were recovered from the train parts or coupons could be kept at room temperature for 3 h and at 4°C for up to a week with minimal changes in concentration (average log_10_ change from −0.07 to 0.05) (Table S2). Based on these results, samples were stored at 4°C for up to a week after collection, and the plaque assays were repeated when the P. syringae lawns did not show strong growth. The efficacy was calculated by subtracting the average log_10_ concentration at any given time point on the coated surfaces from the average log_10_ concentration on the noncoated surfaces. The coating was considered to be effective against ɸ6 if, under any given set of conditions, the log_10_ reduction achieved was ≥3.0. This criterion was based on the US EPA interim guidance for the evaluation of the efficacy of antimicrobial surface coatings, which states that a 3 log_10_ reduction of test microbes within 1 to 2 h contact times is required for product registration (15).

### Application of interfering substances.

To mimic contamination with organic matter, 40 μL of bovine serum albumin (BSA 3 g/L; Sigma-Aldrich) or fetal bovine serum (FBS; Sigma-Aldrich) were pipetted onto coated and noncoated coupons, prior to inoculation with ɸ6. The efficacy was then assessed on the coated and noncoated coupons (with and without interfering substances), as described in the previous section.

Similarly, areas of 48, 45, and 25.5 cm^2^ were drawn out on coated and noncoated tray tables, arm rests, and hand poles, respectively. FBS was applied to each test area (200 μL for the tray tables and the arm rests, and 100 μL for the hand poles) and spread across the whole area using FLOQSwabs (Copan Diagnostics). The train parts were left to dry under ambient laboratory conditions, prior to inoculation with ɸ6. The survival of ɸ6 on coated and noncoated test areas (with and without FBS) was assessed.

### Wiping.

A wiping protocol was developed to simulate the mechanical action of cleaning. J cloths (Chicopee J-Cloth Plus) were cut into 48 cm^2^ swatches and moistened with sterile water. They were then used to wipe the surface of a tray table upwards 5 times and across 5 times (10-wipe protocol) or upwards 20 times and across 20 times (40 wipe protocol; only applied to side A, HPL). Both coated and noncoated tray tables (in the presence and absence of dried FBS) were subjected to wiping. Following the wiping, ɸ6 was inoculated on coated and noncoated test surfaces, and its survival was assessed as described previously. Two experimental trials with three technical replicates per condition were carried out.

### Statistical analysis.

The statistical analysis was done with a regression framework in which the statistical significance threshold was set at 5% and the composite Wald test was used to ascertain the statistical significance. Details about the models, the estimates, and their 95% confidence intervals and *P* values are available in the supplemental material. All of the analyses was carried out in STATA 17.0.
