# Supplementary material for: Laboratory Evaluation of a Quaternary Ammonium Compound-Based Antimicrobial Coating Used in Public Transport during the COVID-19 Pandemic
Source: Appl Environ Microbiol. 2023 Mar 1;89(3):e01744-22. doi: 10.1128/aem.01744-22 (PMC10057021; doi:10.1128/aem.01744-22)
Supplement: Supplemental file 1 — Supplemental material. Download aem.01744-22-s0001.pdf, PDF file, 0.2 MB [file aem.01744-22-s0001.pdf]

Supplemental material

Statistical methods

The outcome in each analysis was the logarithm of the ϕ6 count and tube (with duplicate measurements) was used as the cluster variable. Adherence to model assumptions was investigated by plotting graphs of the residual or standardised residual against each of the explanatory variables and in a quantile-quantile plot in the censored and uncensored experiments respectively.

Validation of storage conditions for ϕ6 suspensions

Triplicate tubes containing ϕ6 in Dey-Engley neutralizing broth (DEB) were set up and periodically enumerated via plaque assays at room temperature (0, 1, 2 and 3 hours) and at 4 °C (0, 24, 48 and 168 hours). The results were analysed using uncensored linear regression with temperature and time as categorical variables (Supplementary table 2).

Efficacy of the antimicrobial coating

Censored linear regression was performed on all experiments assessing the efficacy of the antimicrobial coating. The variables and interactions included on each of the models are shown on supplementary table 1.

Supplemental table 1. Variables and interactions included on the statistical models.

Experiment	Variables	Interactions	Results table
Application of the antimicrobial coating on glass coupons	Application method, date of experiment, contact time	NA	Supplementary table 3
Application of antimicrobial coating on stainless steel and polystyrene coupons	Antimicrobial coating application, days after application of antimicrobial coating, coupon material, contact time	Antimicrobial coating application, days after application of antimicrobial coating, coupon material	Supplementary table 4
Interfering material evaluation with BSA and FBS	Antimicrobial coating application, interfering material, coupon material, contact time	Antimicrobial coating application, interfering material, coupon material	Supplementary table 5
Application of the antimicrobial coating on tray tables and an armrest	Antimicrobial coating application, days after application of antimicrobial coating	Antimicrobial coating application, days after application of antimicrobial coating,	Supplementary table 6

	(co), interfering material, train part, contact time	interfering material, train part	
Application of the antimicrobial coating on a hand pole	Antimicrobial coating application, days after application of antimicrobial coating, interfering material, contact time	Antimicrobial coating application, days after application of antimicrobial coating (co), interfering material	Supplementary table 7
Effect of wiping on efficacy of the antimicrobial coating on a train tray table in the presence of FBS as an interfering material	Antimicrobial coating application, days after application of antimicrobial coating, interfering material, train part, wiping, contact time	Antimicrobial coating application, days after application of antimicrobial coating, interfering material, train part, wiping	Supplementary table 8

NA – Not applicable, BSA – bovine serum albumin, FBS – foetal bovine serum, (co) – continuous variable, categorical otherwise

Where there was a continuous variable in the model, it was entered as a cubic function and successively simplified to a linear function if the highest order term was not statistically significant at each step. After doing this where applicable, each model was simplified by removing the highest order interaction term that was not statistically significant and the largest p-value or was not estimable. If a model was not estimable, the next simplest model was fitted by removal of an interaction if more than one interaction of the same order in the model or the highest order interaction term, as applicable. This process continued until either all the terms in the model were main effects or the highest order interaction(s) found to be statistically significant, together with all its (their) lower order interactions and main effects and the other variables as main effects not involved in any interaction, yielding the final model.

Results

Supplementary tables 2-8 show the estimates, their 95% confidence intervals (CIs) and p-values from the final models. Model diagnostics, as described in the methods, revealed no major violations of modelling assumptions.

Supplemental table 2. Validation of storage conditions for ϕ6 suspensions. There was a statistically significant difference over time in the $\log_{10}$ counts, with time points 2 and 3 hours significantly lower, and time point 48 hours significantly higher, than time point 0. However, the range of average $\log_{10}$ change was from -0.07 to 0.05 which, in the context of these experiments, is not considered of practical significance.

Variable	Category	Coeff.*	95% CI*	p-value
Time (hours)	0	0.00	-	<0.001
	1	-0.01	-0.05, 0.02	
	2	-0.04	-0.08, -0.01	
	3	-0.07	-0.10, -0.03	
	24	0.00	-0.04, 0.03	
	48	0.05	0.02, 0.09	
	168	-0.03	-0.07, 0.00	

*Adjusted for temperature

Supplemental table 3. Application of the antimicrobial coating on glass coupons.
Application of the antimicrobial coating on glass coupons resulted in significantly lower log_{10} counts of ϕ6 than those obtained on non-coated coupons. The two methods of coating did not differ significantly from one another.

Variable	Category	Coeff.*	95% CI*	p-value
Application method	No coating	0.00	-	<0.001
	Manual spraying	-3.30	-4.34, -2.25	
	Pipetting (75µL)	-3.84	-5.55, -2.13	

*Adjusted for date of experiment and contact time (minutes)

Supplemental table 4. Application of antimicrobial coating on stainless steel and polystyrene coupons. Application of antimicrobial coating on stainless steel and polystyrene coupons resulted in significantly lower log_{10} counts of ϕ6 than those obtained on non-coated coupons. No statistically significant differences were found between the materials or results obtained on different number of days since application of the antimicrobial coating.

Variable	Category	Coeff.*	95% CI*	p-value
Antimicrobial coating application	No coating	0.00	-	<0.001
	Coating (Pipetting 40µL)	-6.16	-6.94, -5.38	
Material	Polystyrene	0.00	-	0.4
	Stainless steel	0.27	-0.39, 0.92	

Days after application of antimicrobial coating	1	0.00	-	0.4
	7	0.33	-0.68, 1.34	
	14	0.15	-0.84, 1.14	
	21	0.10	-0.94, 1.13	
	28	1.02	-0.09, 2.13	

*Adjusted for contact time (minutes)

Supplemental table 5. Interfering material evaluation. Application of the antimicrobial coating on stainless steel and polystyrene coupons in the presence or absence of bovine serum albumin (BSA) or fetal bovine serum (FBS) as interfering materials. When FBS was applied over the antimicrobial coating the average log_{10} counts were significantly higher compared to when BSA or no interfering material were applied.However, no difference was found between average log_{10} counts when no interfering substance was present or when BSA was applied.

Variable		Coeff*	95% CI*	p-value
Antimicrobial coating application	Interfering material			
No coating	No material	0.00	-	<0.001[+]
	BSA	0.04	-1.04, 1.11	
	FBS	0.06	-1.01, 1.11	
Coating	No material	-6.86	-7.96, -5.75	
	BSA	-7.00	-8.41, -5.59	
	FBS	-2.11	-3.25, 0.97	

*Adjusted for coupon material and contact time (minutes) [+]For interaction

Supplemental table 6. **Application of the antimicrobial coating on tray tables and an armrest.** The efficacy of the antimicrobial coating was evaluated in the presence or absence of FBS as an interfering material. A significant three-way interaction was found for the train part, antimicrobial coating application and interfering material variables. The average log_{10} reduction associated with the antimicrobial coating was significantly higher on side B compared to side A of the tray table and significantly lower for both sides when FBS was present, with no difference detected between the two materials. The presence of FBS did not significantly affect the average log_{10} counts on tray tables when no antimicrobial coating was present. In comparison with the tray table, average log_{10} counts on the armrest were lower but the application of the antimicrobial coating did not lead to significant reductions on this train part. A further significant interaction between days after application and coating was also found, with the per seven day decrease without a coating significant while that with a coating showed a non-significant increase, although both well within one log_{10}.

These results are likely to reflect the differences in survival of ϕ6 on different experiment days and a slight loss of efficacy of the antimicrobial coating with time.

Variable			Coeff*	95% CI*	p-value
**Train part**	**Antimicrobial coating application**	**Interfering material**			
Tray table (Side B-CSS)	No coating	No material	0.00	-	<0.001^{+}
		FBS	0.12	-0.33, 0.57	
	Coating	No material	-4.32	-5.49, -3.15	
		FBS	-0.67	-1.24, -0.09	
Tray table (Side A- HPL)	No coating	No material	0.06	-0.37, 0.49	
		FBS	0.17	-0.27, 0.61	
	Coating	No material	-2.92	-3.95, -1.88	
		FBS	-0.61	-1.20, -0.02	
Armrest (Terluran 22)	No coating	No material	-1.08	-1.53, -0.63	
		FBS	-0.67	-1.10, -0.24	
	Coating	No material	-0.67	-1.10, -0.24	
		FBS	-1.04	-1.62, -0.47	
**Days after application of antimicrobial coating**		**Antimicrobial coating application**			
4-27 days		No coating	-0.15 per week increase in days after application	-0.25, -0.04	0.001^{+}
		Coating	0.04 per week increase in days after application	-0.12, 0.21	

*Adjusted for time (minutes) $^{+}$For interaction

Supplemental table 7. Application of the antimicrobial coating on a hand pole. The efficacy of the antimicrobial coating was evaluated in the presence or absence of FBS as an interfering material. A significant interaction was found between the antimicrobial coating application and the interfering material. There was a significant reduction on $\log_{10}$ counts when the coating was applied but not when FBS was subsequently applied as an interfering material. FBS did not significantly impact $\log_{10}$ counts when no antimicrobial coating was present. There appeared to be no significant relationship between days after application of the antimicrobial coating and the $\log_{10}$ count.

Variable			Coeff*	95% CI*	p-value
Train part	Antimicrobial coating application	Interfering material			
Hand pole	No coating	No material	0.00	-	<0.001+
		FBS	0.13	-0.65, 0.91	
	Coating	No material	-3.87	-5.30, -2.44	
		FBS	-0.09	-0.82, 0.65	
	**Days after application of antimicrobial coating**				
	7		0.00	-	0.3
	20		0.39	-0.40, 1.17	

*Adjusted for time (minutes) +For interaction

Supplemental table 8. Effect of wiping on efficacy of the antimicrobial coating on a train tray table in the presence of FBS as an interfering material. The impact of wiping depended on whether the material had been coated and whether FBS was present. When FBS was present, wiping did not impact the log_{10} counts. Wiping significantly reduced the average log_{10} counts when the coating was present but there was no interfering material. No significant difference was found for days after application of the antimicrobial coating or between the two sides of the tray table.

Variable			Coeff*	95% CI*	p-value
Antimicrobial coating application	Interfering material	Wiping			
No coating	No material	No wiping	0.00	-	<0.001+
		10 wipes	-0.04	-0.35, 0.27	
		40 wipes	-0.44	-0.83, -0.05	
	FBS	No wiping	0.00	-0.27, 0.27	
		10 wipes	0.07	-0.26, 0.39	
		40 wipes	-0.17	-0.65, 0.31	
Coating	No material	No wiping	-3.38	-4.26, -2.49	
		10 wipes	-1.25	-1.89, -0.60	
		40 wipes	-1.34	-1.86, -0.81	
	FBS	No wiping	-0.10	-0.35, 0.15	
		10 wipes	-0.10	-0.40, 0.20	
		40 wipes	-0.10	-0.40, 0.20	
**Days after application of coating**					
1			0.00	-	0.8
2			0.10	-0.30, 0.51	
9			0.10	-0.46, 0.67	

10<br>11	-0.22<br>-0.16	-0.94, 0.50<br>-0.88, 0.57	
**Train part**			
Tray table (Side B-CSS)<br>Tray table (Side A- HPL)	0.00<br>0.38	-<br>-0.18, 0.94	0.18
